# The role of the l-IPS in the comprehension of reversible and irreversible sentences: an rTMS study

**DOI:** 10.1007/s00429-020-02130-6

**Published:** 2020-08-25

**Authors:** Lorenzo Vercesi, Prerana Sabnis, Chiara Finocchiaro, Luigi Cattaneo, Elena Tonolli, Gabriele Miceli

**Affiliations:** 1 CIMeC-Center for Mind/Brain Sciences, University of Trento, Rovereto, Italy; 2 International Doctorate for Experimental Approaches To Language and Brain (IDEALAB), University of Trento, Rovereto, Italy; 3 DipSCo, Department of Cognitive Sciences, University of Trento, Rovereto, Italy; 4 Centro Linceo Interdisciplinare ‘Beniamino Segre’, Accademia Dei Lincei, Rome, Italy

**Keywords:** rTMS, Thematic role assignment, Semantic reversibility, l-IPS, Sentence processing

## Abstract

Thematic roles can be seen as semantic labels assigned to who/what is taking part in the event denoted by a verb. Encoding thematic relations is crucial for sentence interpretation since it relies on both syntactic and semantic aspects. In previous studies, repetitive transcranial magnetic stimulation (rTMS) over the left inferior intraparietal sulcus (l-IPS) selectively influenced performance accuracy on reversible passive (but not active) sentences. The effect was attributed to the fact that in these sentences the assignment of the agent and theme roles requires re-analysis of the first-pass sentence parsing.

To evaluate the role of reversibility and non-canonical word order (passive voice) on the effect, rTMS was applied over l-IPS during a sentence comprehension task that included reversible and irreversible, active and passive sentences. Participants were asked to identify who/what was performing the action or who/what the action was being performed on.

Stimulation of the l-IPS increased response time on reversible passive sentences but not on reversible active sentences. Importantly, no effect was found on irreversible sentences, irrespective of sentence diathesis.

Results suggest that neither reversibility nor sentence diathesis alone are responsible for the effect and that the effect is likely to be triggered/constrained by a combination of semantic reversibility and non-canonical word order. Combined with the results of previous studies, and irrespective of the specific role of each feature, these findings support the view that the l-IPS is critically involved in the assignment of thematic roles in reversible sentences.

## Introduction

### Thematic role assignment and sentence comprehension

In all languages, sentence comprehension requires information at different levels to be computed and integrated. Regardless of the language spoken by an individual, correctly establishing who/what is taking which part in the event described by the verb is essential for sentence interpretation. To understand a sentence like *The girl watches the tree*‚ for example, the listener must identify *the girl* as the one who is doing the action (the agent), and *the tree* as what the action is being performed on (the patient/theme). This process requires the integration of syntactic and semantic information.

Word order is an important syntactic dimension in sentence comprehension. In S-V-O languages like Italian and English, words usually appear in canonical order in active sentences (agent-verb-theme), and in non-canonical order in passive sentences (theme-verb-agent). Therefore, the processing of passive sentences is more demanding. Unsurprisingly, children learn to produce passives later than actives (e.g., Kirby 2010). Evidence that passive sentences pose a greater computational load than active sentences has been provided by several studies (see for example Ferreira (2003); Meyer et al. 2012). Processing cost in passives is increased by the fact that, due to the presence of the auxiliary verb and of the by-phrase, they are also longer and structurally more complex than actives. Finally, sentences in the passive voice are used less frequently than active sentences. In languages with an S-V-O structure, in active sentence the syntactic role of the subject always matches the semantic role of the agent (subject = agent), so that one can apply a rule according to which the first constituent corresponds to the role of agent; in passive sentences, instead, there is a mismatch between syntactic and semantic roles (subject ≠ agent = theme; object ≠ theme = agent). This means that in all languages with an S-V-O structure the systematic rules driving the assignment of thematic roles are equivalent. English and Italian, for example, share the same S-V-O structure, so they are completely interchangeable.

Sentence comprehension is also modulated by semantic dimensions. In semantically irreversible sentences, only one constituent can be the agent. In the active sentence *La ragazza guarda l’albero* (*The girl watches the tree*), word order and semantic constraints greatly facilitate thematic role assignment. In the corresponding passive sentence *L’albero è guardato dalla ragazza* (*The tree is watched by the girl*)‚ non-canonical word order makes role assignment less easy. In this case, agent and theme roles can still be assigned based solely on semantic knowledge. In semantically reversible sentences, however, both constituents can be agent or theme. Therefore, comprehension requires syntactic processing. In active sentences (*La ragazza bacia il ragazzo*—*The girl kisses the boy*), canonical word order facilitates thematic role assignment. In passive sentences (*Il ragazzo è baciato dalla ragazza*—*The boy is kissed by the girl*), however, word order is non-canonical and semantic knowledge cannot constrain thematic role assignment. Due to non-canonical word order and semantic reversibility, these sentences must be re-analysed and thematic roles re-assigned (Chomsky 1965, 1981; Pollard and Sag 1994; Bresnan 2000).

### The neural basis of thematic role assignment

The neuroanatomical bases of sentence comprehension, particularly of passive and semantically reversible sentences, have been investigated in several studies, focusing on both normal and clinical populations, as well as involving different techniques (lesion studies via Voxel-based Lesion Symptom Mapping (VLSM), fMRI studies, and TMS studies in neurologically intact volunteers). For a recent review of neuroimaging data see Walenski et al. (2019).

Historically, the neural mechanisms underlying the comprehension of reversible sentences were first investigated indirectly in studies focusing on language disorders of aphasic patients (see for example Caramazza and Zurif 1976; Caplan and Futter 1986; Grodzinsky 2000; Love et al. 2008; Thompson and Choy 2009). The inability to comprehend and produce sentences as a consequence of the inability to map thematic roles onto syntactic roles and vice versa was reported in two individuals with aphasia suffering from damage to left parieto-temporal regions (Caramazza and Miceli 1991; Martin and Blossom-Stach 1986). These early, almost anecdotal observations have been replicated by more systematic investigations. Thothathiri et al. (2012) used VLSM (Bates et al. 2003) in a large group of aphasic participants. Poor comprehension of reversible sentences, resulting in role reversal errors, correlated significantly with damage to the left temporo-parietal cortex, but not with lesions in the inferior frontal gyrus. These results are consistent with other VLSM studies (see Dronkers et al. 2004 or Bates et al. 2003) and with an investigation of PET metabolism in patients with aphasia, that found a correlation between parieto-temporal damage and the comprehension of sentences of varying syntactic complexity (Caplan et al. 2007, 2015). Evidence from these studies is consistent with the hypothesis that parieto-temporal areas are critical for the comprehension of reversible sentences, perhaps because they are involved in thematic labeling in non-canonical sentences, such as passive declaratives. Similar results were reported by Rogalsky et al. (2018), who studied the comprehension of canonical and non-canonical reversible sentences in patients with chronic focal cerebral damage through a VLSM approach. They found maximal overlap in left posterior superior temporal and inferior parietal regions.

A number of neuroimaging investigations on cognitively unimpaired participants provides converging evidence (for reviews see Meyer and Friederici 2015; Rodd et al. 2015; Martin et al. 2015; Walenski et al. 2019). For instance, an fMRI study by Richardson et al. (2010) evaluated the impact of semantic reversibility on the comprehension of semantically reversible sentences over a range of syntactic structures. The contrast between reversible and irreversible sentences showed activation in a lateral portion of the left posterior-superior temporal gyrus and in an inferior parietal region.

Neuromodulation studies provide additional support for the role of left parietal regions in the comprehension of reversible sentences. Finocchiaro et al. (2015) delivered transcranial magnetic stimulation (rTMS) to three sites along the l-IPS, in order to investigate their contribution to thematic role assignment during a sentence comprehension task. Experimental stimuli consisted of active and passive, semantically reversible sentences. In agreement with predictions, rTMS to the posterior l-IPS site selectively increased performance accuracy on reversible passives, while leaving performance on reversible actives unaffected.

The increasing evidence in support of the involvement of inferior parietal regions in the assignment of thematic roles raises fundamental questions on the functional role of the inferior parietal lobe in sentence comprehension. The aim of the present work is to shed light on the specific contribution of this region to sentence comprehension and more specifically to thematic role mapping.

### Experiment and hypotheses

The results in Finocchiaro et al. converge with available lesion data and neuroimaging investigations in supporting the idea that parietal regions are critical for the comprehension of reversible sentences. However, the study’s experimental stimuli included only reversible sentences. This leaves the mechanisms underlying the observed effect unclear. Thematic re-analysis is required for sentences in which syntactic cues (word order) or semantic cues (reversibility) are insufficient to constrain thematic role assignment. This is the case for reversible passives. In these sentences, however, re-analysis could be triggered and/or constrained by several features of the stimulus. To establish whether passive diathesis, semantic reversibility, or both are involved in re-analysis, and to analyze in greater detail the role of l-IPS in sentence comprehension, reversible and irreversible, active and passive stimuli were included in the present rTMS study.

During a sentence comprehension task, focal rTMS was delivered online to the posterior part of l-IPS. This site was selected because in Finocchiaro et al. (2015) only stimulation of this portion affected performance accuracy on reversible passives, whereas rTMS on anterior and middle l-IPS sites had no effect. In addition, lesion and neuroimaging studies converge in assigning this region a role in the comprehension of reversible sentences. Stimuli were organized in a 2 × 2 design: sentences could be either active or passive, and reversible or irreversible. We wished to understand whether the effect is due to passive diathesis or semantic reversibility per se, or to a combination of the two. If the effect were driven by reversibility, both active and passive reversibles should be affected by rTMS; on the contrary, if diathesis per se were relevant, the effect should involve passive sentences regardless of semantic reversibility.

## Materials and methods

### Materials

A sample of 136 sentences was prepared. Of these sentences, 120 were used as experimental stimuli; the remaining 16 served as practice items. The following procedure was used in stimulus preparation.

A preliminary set of sentences was created. Sentences included commonly used nouns (e.g. *architetto*, architect, *bambina*, girl) and verbs (e.g., *colpire*, hit) that could be used in both reversible and irreversible stimuli. The sentences presented during the experimental procedure contained 30 verbs. Each verb was paired with two sets of two nouns. In one set both nouns were animate, while in the other set a noun was animate and the other inanimate. As a result, each verb was used to generate two reversible and two irreversible sentences (one active, one passive). Hence, four types of sentences were prepared: reversible active sentences (RA), reversible passive sentences (RP), irreversible active sentences (IA), and irreversible passive sentences (IP). Examples of each sentence type are presented in Table 1.

**Table 1:** Examples of sentence types included in the sample

Sentence Type	Example
Reversible Active Sentences (RA)	*La cameriera ha trovato la bambina* (The maid found the girl)
Reversible Passive Sentences (RP)	*La bambina è stata trovata dalla cameriera* (The girl was found by the maid)
Irreversible Active Sentences (IA)	*La cameriera ha trovato la borsa* (The maid found the purse)
Irreversible Passive Sentences (IP)	*La borsa è stata trovata dalla cameriera* (The purse was found by the maid)

Word frequency was controlled based on an Italian corpus (Bertinetto et al. 2005). Raw frequency values were used to obtain log frequencies before finalizing the list, to exclude extremely frequent or infrequent verbs/nouns. The log frequency of words in reversible and irreversible sentences was comparable. Word length was also balanced across reversible and irreversible sentences. Given the presence of the auxiliary verb and of the *by*-phrase, passive sentences were systematically 2 syllables longer than actives. In a pilot study, reversible and irreversible sentences were comparable for response times and performance accuracy.

A training procedure was administered before the actual experiment. Training sentences included four verbs, each of which was presented in the four contexts (RA, RP, IA and IP).

### Participants

To establish the numerosity of our sample, we estimated an "ideal" sample size based on previous data in the relevant literature. The ideal number of participants was 14 for *α* = 0.05 and ten for *α* = 0.01. Since we adopted a within-participants design, in which all participants took part in two experimental sessions, we decided to set our sample size at 24. We also calculated the size of our effect based on the data we collected. In particular, we compared means of real vs sham significant post hoc contrasts (Cohen's *d* = 0.5, Glass's *delta* = 0.43 and Hedges's *d* = 0.53).

24 native Italian speakers (healthy and right-handed) were tested (*f* = 15, *m* = 9; mean age = 24.70, SD = 2.34). All participants were students from the University of Trento. They had unimpaired or corrected-to-normal vision, and had no prior history of neurological conditions, seizures, or psychiatric symptoms. For each participant, a structural MRI was used to accurately identify the area that would be targeted by rTMS stimulation. To establish eligibility for the rTMS study, each participant completed a safety questionnaire. All participants read and signed a consent form before the experiment started. The testing protocol was authorized by the Ethical Committee of the University of Trento.

### Procedure

Each participant took part in two experimental sessions, spaced by 1 week. Overall, the experiment required ~ 1.5 h per participant.

During the experiment, participants were seated in front of a computer screen. They were asked to read a sentence presented at the center of the screen and to answer the question presented at the beginning of each block by pressing one of two keys on the computer keyboard (2-alternative, forced choice task). The experiment was run on the E-Prime software.

Six blocks with the identical structure were created. Two served as practice, while the remaining four were the experimental blocks.

At the beginning of each block participants were presented with one of the following instructions written on the computer screen:

Instruction A: *Identify who is DOING the action* (agent question).

Instruction B: *Identify who/what is RECEIVING* the action (theme question).

Each instruction applied to an entire sentence block (*n* = 30). Participants were asked to identify the agent in one half of the blocks, and the theme in the other half (see Table 2 for examples). Task demands were diversified across blocks to reduce the likelihood that participants would develop a systematic strategy. For example, if all blocks had been of the ‘identify-the-agent’ type, the correct response to irreversible stimuli could be obtained by systematically looking for the inanimate constituent that would appear to the right of the verb in actives (“***La bambina mangia la mela***—***The girl eats the apple”***) and to the left of the verb in passives (“*La mela è mangiate dalla bambina*—*The apple is eaten by the girl*”). Blocks were counterbalanced so that two ‘agent’ or two ‘theme’ blocks were not administered consecutively (ABBA or BAAB). The same stimuli were presented in the real and sham conditions.

**Table 2:** Examples of trials for both the agent-question and the theme-question

Question	Sentence	Agent	Theme	Correct answer	
Who was doing the action (agent)	The maid found the purse	The maid	The purse	The maid	
Who/what was receiving the action (theme)	The doctor found the boy	The doctor	The boy	The boy	

Accuracy and RT measures were also collected.

Four experimental blocks were prepared, each containing 30 sentences. Half of the sentences in each block were active and the other half were passive. Each block had the following structure:

30 sentences:

- 15 active sentences (alternately seven or eight reversible)

- 15 passive sentences (alternately eight or seven irreversible)

**Fig. 1:**
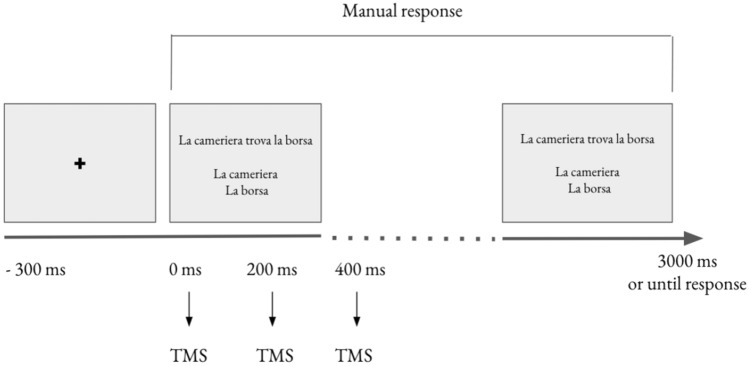
Timeline of the experiment

Trials in each block were randomized and counterbalanced across participants. As mentioned above, participants had a fixed response window of 3000 ms. First, all responses were standardized by converting RT scores into *z scores.* For RT analyses, responses that exceeded this temporal window, as well as responses faster or slower than the mean RT of each subject by ≥ 2 SD, were excluded. Overall, 4.3% of the responses were excluded. For performance accuracy, responses that exceeded the fixed temporal window were excluded from the analyses. (Fig. 1 shows the experiment timeline).

### TMS protocol

The experiment was run in two conditions: real-TMS and sham-TMS. Each participant completed both conditions. The order of sessions (sham, real vs real, sham) was counterbalanced across participants. Participants received three biphasic pulses at 5 Hz starting from stimuli onset via a MC-B70 butterfly coil and a MagPro Compact stimulator (MagVenture). Hence, pulses were administered at stimulus onset (0 ms), at 200 ms, and at 400 ms (see Fig. 1). The sham-TMS was administered by inserting a spacer between the TMS coil and the scalp. Before starting the experiment, the individual visible resting motor threshold (RMT) was calculated as the lowest stimulation intensity applied over the primary motor cortex which produces more than five visible twitches of the right hand out of ten stimuli. Stimulation intensity during the experiment was set at 90% of the individual visible resting state motor threshold (RMT).

The stimulator was triggered by the E-Prime software through the parallel port. TMS was delivered in an event-related fashion, time-locked to the presentation of visual stimuli.

### MRI co-registration and 3D reconstruction

**Fig. 2:**
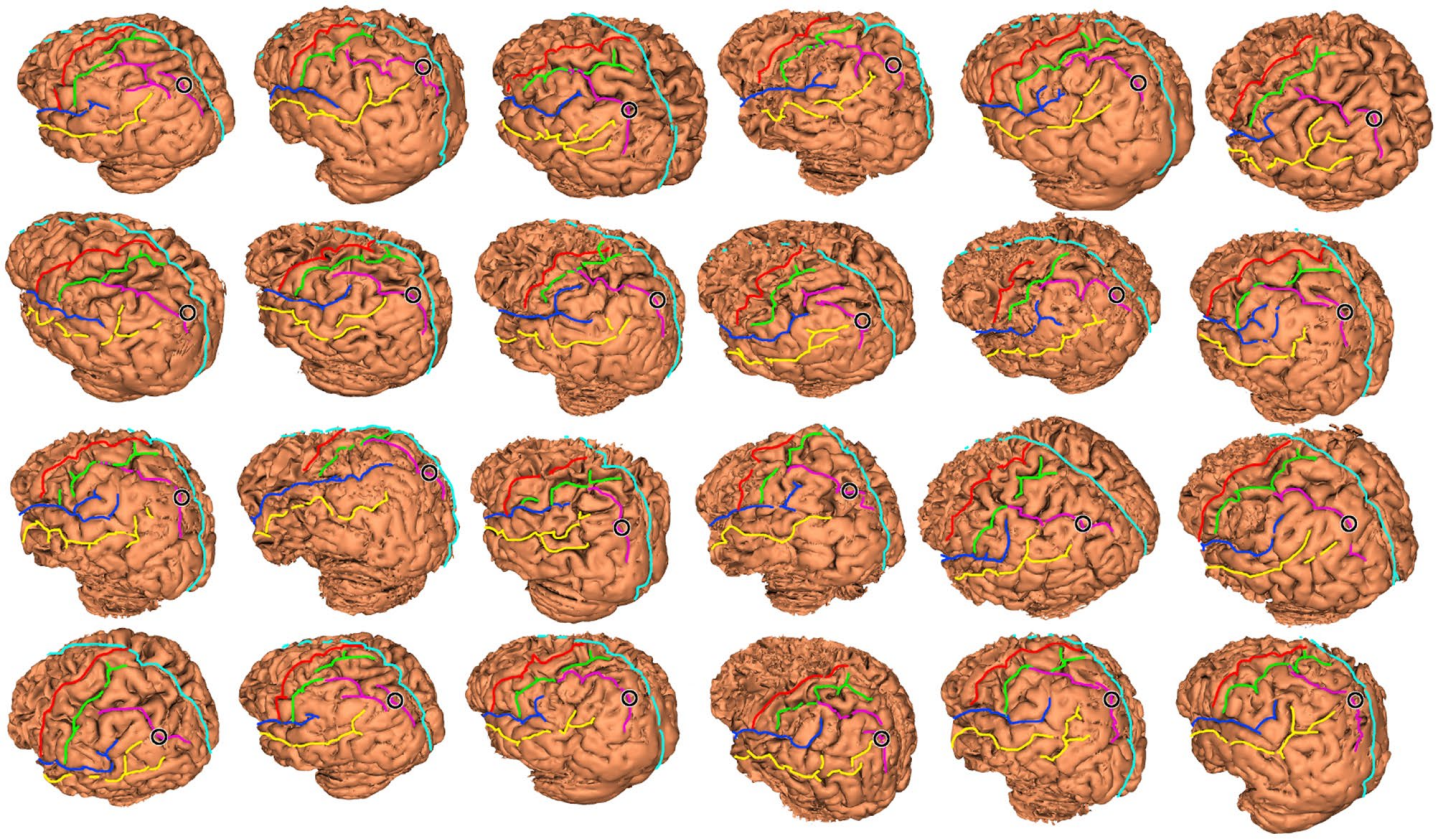
Anatomy and target points (indicated by a circle) for all participants (*n* = 24)

A structural MRI of each participant was available for spatially accurate administration of TMS. Structural images (T1 sequences) were previously acquired through a Prisma Siemens 3 T MRI scan. Stimulation sites were identified on individual 3D brain reconstructions based on macroanatomical landmarks. Before each session, the participant's head, the TMS coil, and the 3D reconstruction of brain and scalp from individual MRI images were co-registered in space by means of the Softaxic Neuronavigation System using a Polaris Spectra camera. Coil position was checked online via the Softaxic Neuronavigation System and was adjusted to the target location based on reconstructions of individual brain anatomy. To locate the spot to be stimulated, the intraparietal sulcus was identified and its length was divided into three segments. Subsequently, the midpoint of the most posterior segment was marked as the TMS target (Fig. 2 shows the anatomy and target points for all subjects).

**Fig. 3:**
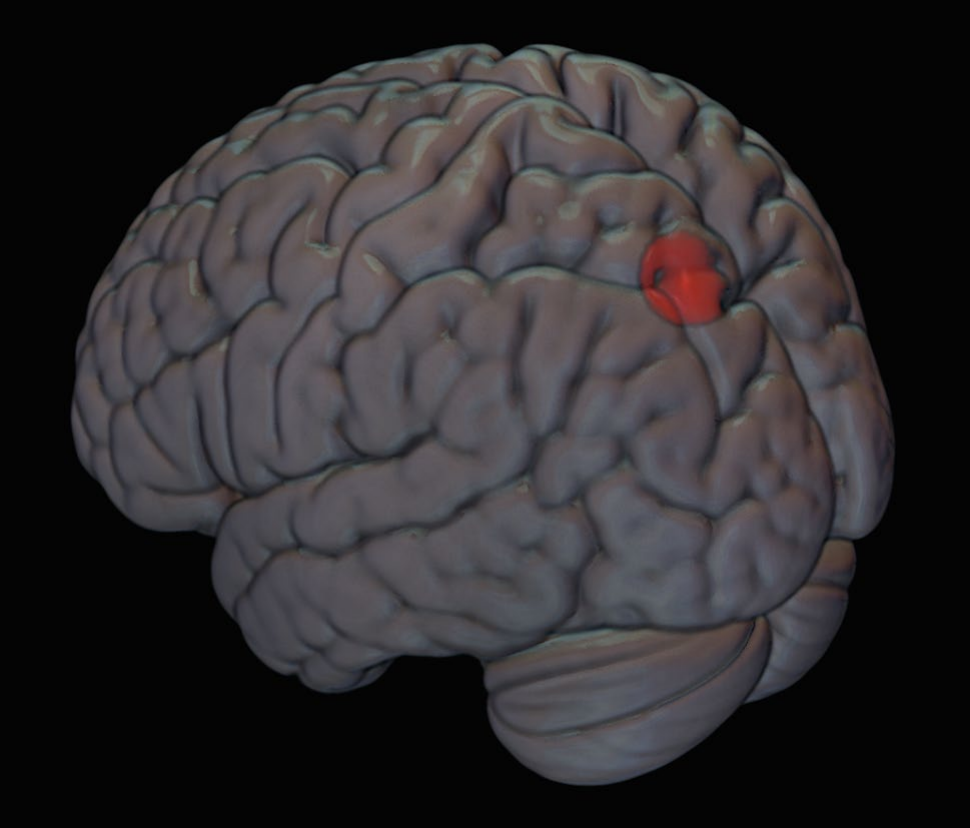
Mean stimulation point across participants (template: spm152). MNI coordinates: *X* = − 22, *Y* = − 79, *Z* = 53)

For each participant, we also compared the localisations of the stimulation sites based on the 3D reconstructions of individual MRIs (native space) to the corresponding stimulation targets based on MNI coordinates. Mean coordinates on the MNI space corresponded to: *x* = − 22.2 (SD =  ± 4.13); *y* = − 79.8 (SD =  ± 3,06); *z* = 53.2 (SD =  ± 4,13). Table 3 shows the MNI coordinates for each participant. Figure 3 shows the mean stimulation point across participants, plotted on a template (spm152).

**Table 3:** Mean TMS coordinates in MNI space for each participant

MNI coordinates			
Subject	*X*	*Y*	*Z*
1	− 26	− 86	46
2	− 14	− 82	52
3	− 20	− 80	48
4	− 26	− 82	46
5	− 24	− 78	56
6	− 20	− 80	52
7	− 18	− 78	58
8	− 22	− 82	50
9	− 26	− 76	52
10	− 18	− 80	58
11	− 24	− 76	60
12	− 24	− 78	58
13	− 22	− 80	50
14	− 22	− 86	46
15	− 26	− 80	54
16	− 18	− 82	52
17	− 20	− 84	52
18	− 26	− 78	56
19	− 32	− 78	56
20	− 21	− 76	54
21	− 18	− 78	58
22	− 28	− 74	56
23	− 17	− 79	56
24	− 21	− 82	51
Mean	− 22.2	− 79.8	53.2
SD	4.13	3.06	4.13

**Fig. 4:**
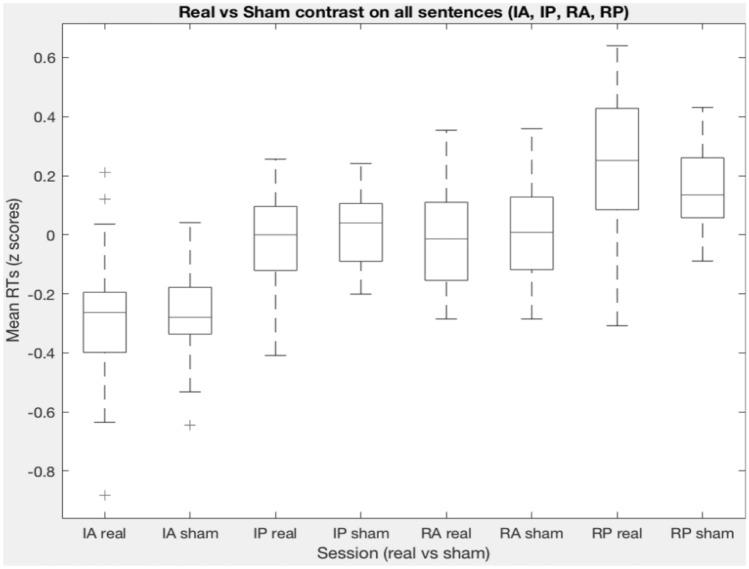
Boxplot of RTs in the Real vs Sham contrast for all experimental conditions (IA, IP, RA, RP)

## Results

### Statistical analyses: RTs

Statistical analyses were run on the sham vs TMS contrast for each experimental condition (irreversible active (IA), irreversible passive (IP), reversible active (RA) and reversible passive (RP)). Table 4 compares descriptive statistics for the sham and TMS contrast in all experimental conditions.

**Table 4:** Descriptive statistics in the sham and TMS contrast for all experimental condition (IA, IP, RA, RP)

	IA_real	IA_sham	IP_real	IP_sham	RA_real	RA_sham	RP_real	RP_sham
Valid	24	24	24	24	24	24	24	24
Missing	0	0	0	0	0	0	0	0
Mean	− 0.26865	− 0.2760	-0.02471	0.02267	− 0.02725	0.005083	0.2488	0.1550
SD	0.2444	0.1505	0.1678	0.1275	0.1700	0.1652	0.2623	0.1509

The experiment followed a 2 × 2 × 2 design: *Stimulation* (Real vs. Sham); *Diathesis* (Active vs. Passive); *Reversibility* (Reversible vs. Irreversible).

To study the effect of stimulation on response times, we used linear mixed-effects regression (LMER). Analyses were performed on *jamovi* (version 1.2) (The jamovi project, 2020; retrieved from: https://www.jamovi.org/). The *General analyses for linear models (GAMLj)* jamovi module was used (Gallucci M., 2019; retrieved from https://gamlj.github.io/). In our model, we wanted to see how differences in RT scores in *STIMULATION* (real-TMS *vs* sham-TMS) were able to explain differences in *DIATHESIS* (active *vs* passive) and *REVERSIBILITY* (reversible *vs* irreversible), taking into account the amount of inter-subject variability in these differences.

**Fig. 5:**
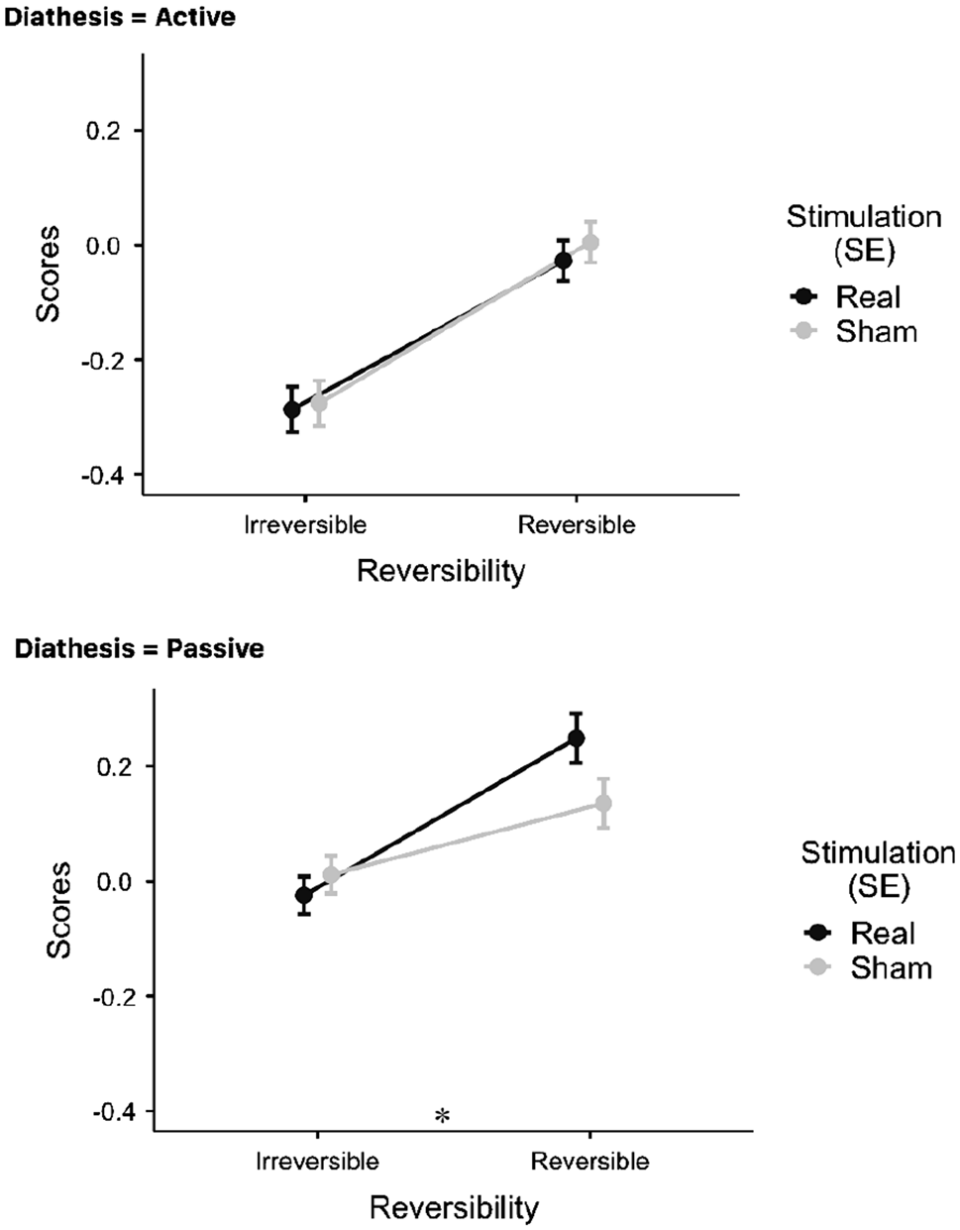
Real vs Sham contrasts for irreversible and reversible sentences (RT scores)

Results showed a main effect of *DIATHESIS* (*F*(1, 23) = 44.379, *p* < 0.001) and *REVERSIBILITY* (*F*(1, 23) = 30.880, *p* < 0.001). No main effect of *STIMULATION* was found ((*F*(1, 138) = 0.167, *p* = 0.683). The effects found on all 2-way interactions failed to reach significance: *DIATHESIS* × *REVERSIBILITY* (*F*(1, 138) = 2.936, *p* = 0.089), *DIATHESIS* × *STIMULATION* (*F*(1, 138) = 2.064, *p* = 0.153) and *REVERSIBILITY* × *STIMULATION F*(1, 138) = 2.349, *p* = 0.128). Remarkably, a 3-way interaction of all the factors *DIATHESIS* × *STIMULATION* × *REVERSIBILITY* was found *F*(1, 138) = 4.223, *p* = 0.042), showing that stimulation significantly influenced response times (see Fig. 4). To further explore this effect, Bonferroni’s Post Hoc Comparisons were performed (see Table 5). These contrasts showed that stimulation affected response speed only on reversible passive sentences (RP). In particular, TMS increased RT on RPs, while having no affect on performance during reversible active sentences (RA) (Fig. 5). No effect of stimulation was found on irreversible sentences (IA, IP).

**Table 5:** Bonferroni’s Post Hoc Comparisons for all the real-TMS vs sham-TMS contrasts

Comparison										
Stimulation	Diathesis	Reversibility	Stimulation	Diathesis	Reversibility	Difference	SE	*t*	df	Pbonferroni
Sham	Active	Irreversible	Sham	Active	Reversible	− 0.28108	0.0555	− 5.0604	62.4	< .001
Sham	Active	Irreversible	Sham	Passive	Irreversible	− 0.28763	0.0509	− 5.6491	77.4	< .001
Sham	Active	Irreversible	Sham	Passive	Reversible	− 0.41150	0.0707	− 5.8201	33.4	< .001
Sham	Active	Irreversible	Real	Active	Reversible	− 0.24875	0.0555	− 4.4783	62.4	< .001
Sham	Active	Irreversible	Real	Passive	Irreversible	− 0.25129	0.0509	− 4.9355	77.4	< .001
Sham	Active	Irreversible	Real	Passive	Reversible	− 0.52483	0.0707	− 7.4230	33.4	< .001
Sham	Active	Reversible	Sham	Passive	Reversible	− 0.13042	0.0509	− 2.5615	77.4	0. 346
Sham	Active	Reversible	Real	Passive	Reversible	− 0.24375	0.0509	− 4.7874	77.4	< .001
Sham	Passive	Irreversible	Sham	Active	Reversible	0.00654	0.0536	0.1220	45.6	1.000
Sham	Passive	Irreversible	Sham	Passive	Reversible	− 0.12387	0.0555	− 2.2301	62.4	0. 822
Sham	Passive	Irreversible	Real	Active	Reversible	0.03888	0.0536	0.7250	45.6	1.000
Real	Passive	Irreversible	Real	Passive	Reversible	− 0.23721	0.0555	− 4.2705	62.4	0.02
Real	Active	Irreversible	Sham	Active	Irreversible	− 0.01054	0.0417	− 0.2527	115.0	1.000
Real	Active	Irreversible	Sham	Active	Reversible	− 0.29162	0.0555	− 5.2502	62.4	< .001
Real	Active	Irreversible	Sham	Passive	Irreversible	− 0.029162	0.0509	− 5.8562	77.4	< .001
Real	Active	Irreversible	Sham	Passive	Reversible	− 0.29817	0.0707	− 5.9691	33.4	< .001
Real	Active	Irreversible	Real	Active	Reversible	− 0.42204	0.0555	− 4.6681	62.4	< .001
Real	Active	Irreversible	Real	Passive	Irreversible	− 0.25929	0.0509	− 5.1425	77.4	< .001
Real	Active	Irreversible	Real	Passive	Reversible	− 0.26183	0.0707	− 7.5721	33.4	< .001
Real	Active	Reversible	Sham	Active	Reversible	− 0.53538	0.0417	− 0.7750	115.0	1.000
Real	Active	Reversible	Sham	Passive	Reversible	− 0.03233	0.0509	− 3.1965	77.4	56
Real	Active	Reversible	Real	Passive	Reversible	− 0.16275	0.0509	− 5.4224	77.4	< .001
Real	Passive	Irreversible	Sham	Active	Reversible	− 0.27608	0.0536	− 0.5556	45.6	1.000
Real	Passive	Irreversible	Sham	Passive	Irreversible	− 0.02979	0.0417	− 0.8709	115.0	1.000
Real	Passive	Irreversible	Sham	Passive	Reversible	− 0.03633	0.0555	− 2.8842	62.4	151
Real	Passive	Irreversible	Real	Active	Reversible	− 0.16021	0.0536	0.0474	45.6	1.000
Real	Active	Irreversible	Real	Passive	Reversible	0.00254	0.0555	− 4.9246	62.4	< .001
Real	Active	Reversible	Sham	Passive	Reversible	0.11333	0.0417	2.7166	115.0	0.231

### Statistical analyses: accuracy

**Fig. 6:**
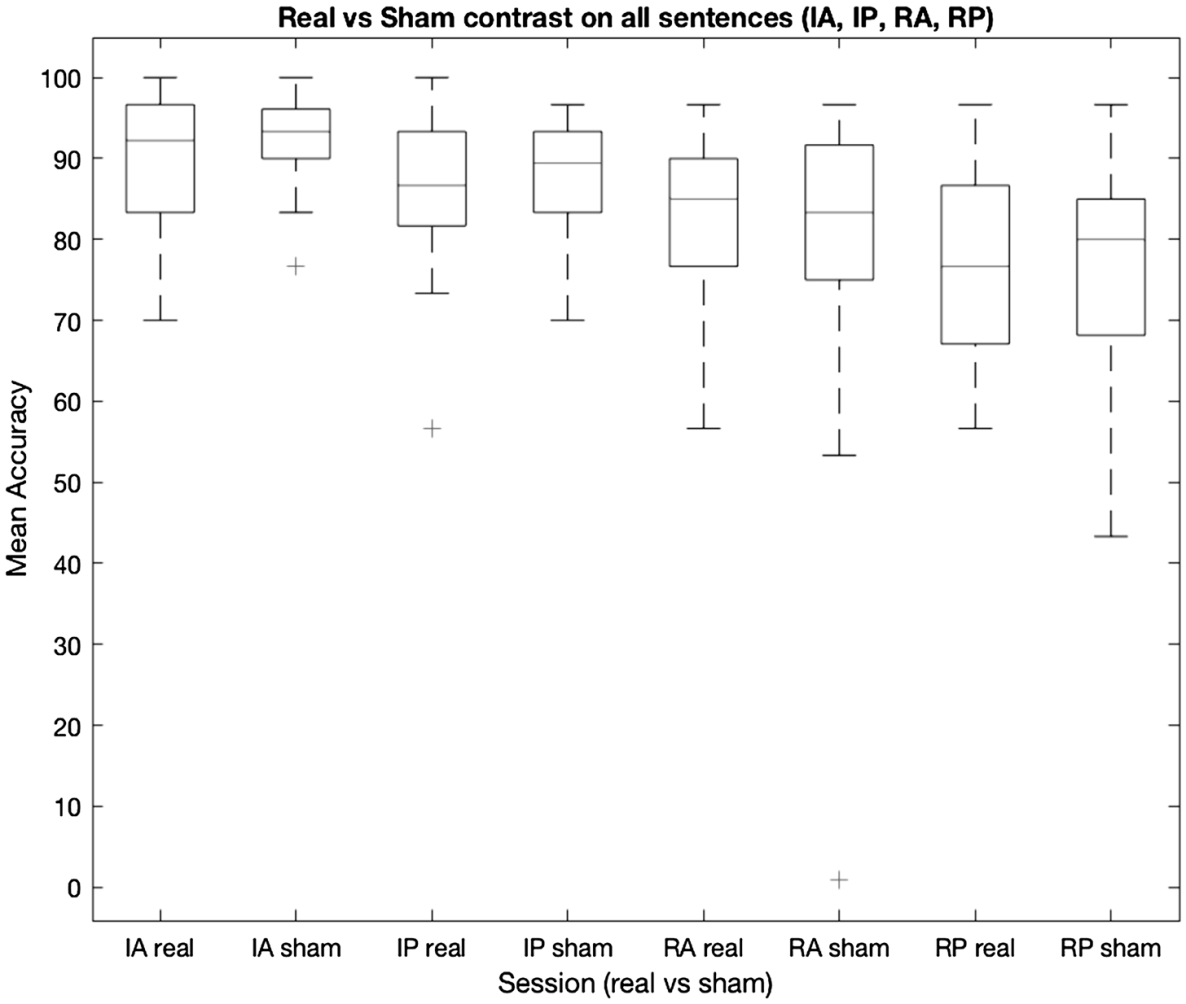
Boxplot of performance accuracy in the Real vs Sham contrast for all experimental conditions (IA, IP, RA, RP)

**Fig. 7:**
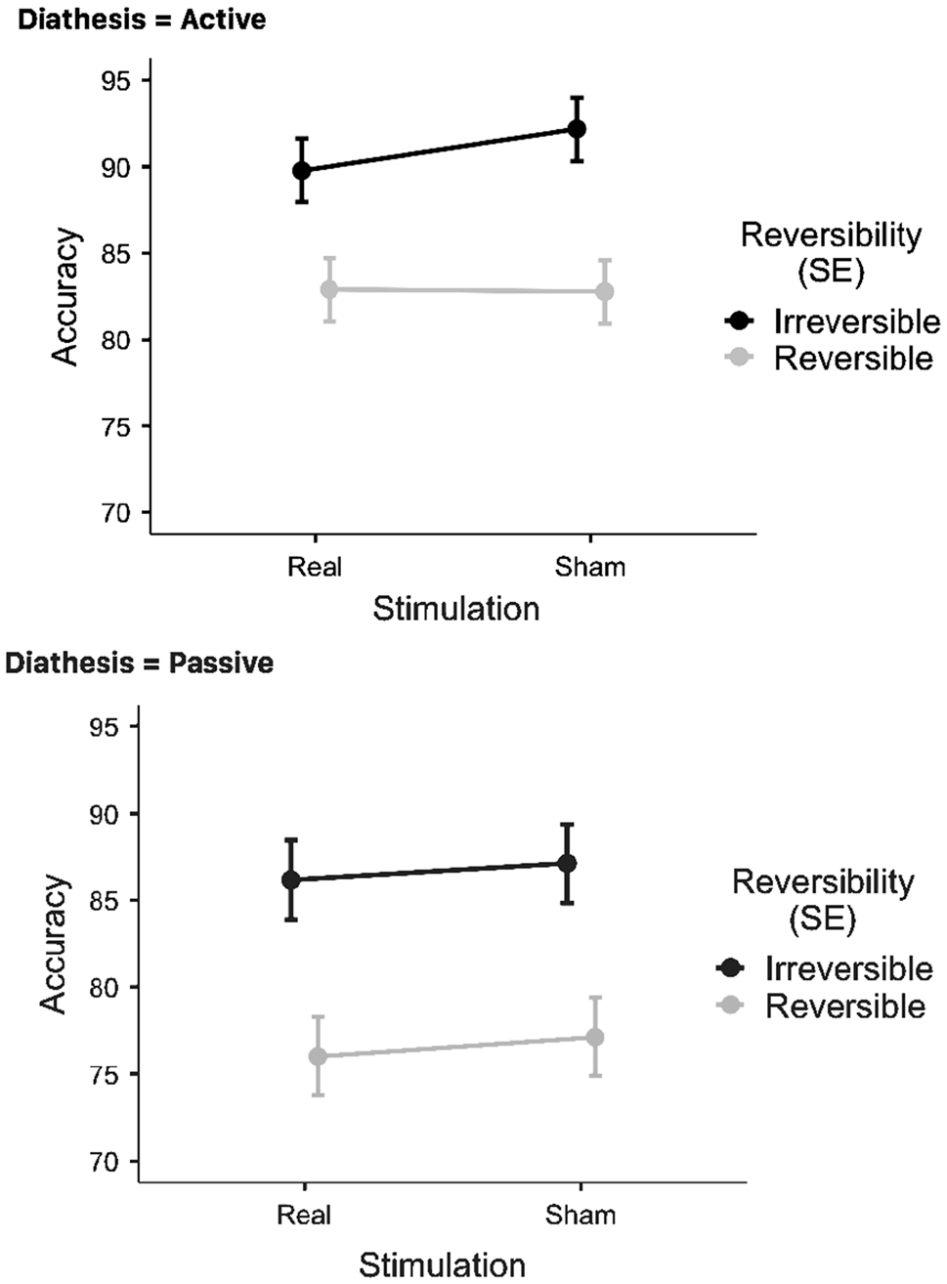
Real vs Sham contrasts for irreversible and reversible sentences (performance accuracy scores)

Performance accuracy was analyzed in the same way as response times. Table 6 compares descriptive statistics for the sham and TMS contrast in all experimental conditions. Overall, stimulation did not affect performance accuracy in either experimental condition. Significant main effects of *DIATHESIS* (*F*(1,40.1) = 18.51640, *p* < 0.001), and *REVERSIBILITY* (*F*(1,161) = 74.88433, *p* < 0.001) were observed. In addition, no interaction effects were found: *DIATHESIS* × *REVERSIBILITY* (*F*(1,161) = 0.82296, *p* = 0.366), *DIATHESIS* × *STIMULATION* (*F*(1,161) = 0.00241, *p* = 0.961), *REVERSIBILITY* × *STIMULATION* (*F*(1,161) = 0.31636, *p* = 0.575), *DIATHESIS* × *STIMULATION* × *REVERSIBILITY* (*F*(1,161) = 0.39822, *p* = 0.529). See Figs. 6 and 7 for a graphical presentation of the results.

**Table 6:** Descriptive statistics in the sham and TMS contrast for all experimental condition (IA, IP, RA, RP)

Descriptives	Stimulation	Diathesis	Reversibility	Accuracy
Mean	Real	Active	Irreversible	89.8
			Reversible	82.9
		Passive	Irreversible	86.2
			Reversible	76.0
	Sham	Active	Irreversible	92.2
			Reversible	82.8
		Passive	Irreversible	87.1
			Reversible	77.1
SD	Real	Active	Irreversible	8.28
			Reversible	9.51
		Passive	Irreversible	9.48
			Reversible	11.04
	Sham	Active	Irreversible	5.34
			Reversible	10.04
		Passive	Irreversible	9.76
			Reversible	15.00

## Discussion

Functional neuroimaging and lesion studies show that parietal and temporal regions are critical for the comprehension of reversible sentences. Neuromodulation studies also show that reversible sentences in passive diathesis behave differently from reversible active sentences. It is commonly assumed that this is because these sentences require thematic reanalysis, i.e., the process through which previously assigned thematic roles are re-mapped as a consequence of non-canonical word order and semantic reversibility (Chomsky 1965, 1981; Pollard and Sag 1994; Bresnan 2000). On this premise, we wished to understand which dimension of reversible passives is the most likely cause of their distinct behavior and, consequently, what is the role of the l-IPS in subsuming the ‘distinctiveness’ of these sentences. We focused on two features of reversible passives that might account for their peculiarity: (i) passive diathesis and (ii) semantic reversibility. Both features have been shown to modulate sentence comprehension and meaning interpretation (Brookshire and Nicholas 1980; Ferreira 2003; Meyer et al. 2012), but their relative weight is uncertain. In our experiment rTMS was selectively delivered to the posterior portion of l-IPS while participants were engaged in a sentence comprehension task that required thematic role assignment in active and passive, reversible and irreversible sentences.

Reversible passive sentences behaved differently from the other sentence types. rTMS over the l-IPS during the comprehension of simple declarative sentences increased response times only on reversible passives while leaving unaffected the performance on active reversible sentences, as well as on active and passive irreversible sentences. Whereas performance accuracy was not influenced by stimulation in any experimental condition, RTs analysis showed that reversible passive sentences behaved differently from the other sentence types. This finding has clear implications. First of all, passive diathesis alone cannot account for the peculiar behavior of reversible passives. If that were the case, rTMS should have influenced performance on all passives, irrespective of reversibility. The same conclusion can be drawn for semantic reversibility: if the effect were due to reversibility, rTMS should have affected only reversible sentences, regardless of whether they were active or passive. A more likely account of the behavioral results, then, is that only the interaction, or the co-occurrence of passive voice and semantic reversibility, triggers re-analysis of thematic role assignment and results in the observed selective effect of rTMS on reversible passive sentences.

On this account, the l-IPS would contribute to the comprehension of reversible sentences by providing the neural substrate needed to solve the computational problems posed by the lack of straightforward syntactic and semantic cues for sentence interpretation. This view is supported by evidence linking the l-IPS to the comprehension of reversible and passive sentences (Keller Carpenter and Just 2001; Wang et al. 2016; Mirman and Graziano 2012; Wu, Waller and Chatterjee 2007; Finocchiaro et al. 2015see also references in the Introduction). Several mechanisms underlying the involvement of l-IPS can be considered.

 The l-IPS could be critical when assigning thematic roles in reversible passive sentences because it represents language-specific knowledge. It would implement both the knowledge necessary for thematic mapping and for sentence voice processing. When comprehension requires integrating both types of linguistic knowledge, as in sentences that are both reversible and passive, the need for re-analysis pushes this area to a critical computational limit and makes it sensitive to rTMS. However, the view that the l-IPS plays a strictly ‘linguistic’ role, even though fully compatible with the observed results, is not entirely consistent with functional considerations and neural observations. From the processing viewpoint, it is unlikely that the comprehension of a passive reversible sentence can be accomplished without involving working memory resources in addition to linguistic knowledge. Furthermore, neurofunctional evidence does not provide unambiguous support for the role of l-IPS in linguistic processes (for reviews see Meyer and Friederici, 2015; Rodd et al. 2015; Martin et al. 2015; Walenski et al. 2019; see also Keller, Carpenter and Just 2001; Richardson et al. 2010; Thothathiri et al. 2012 and Rogalsky et al. 2018).

From an alternative view, the l-IPS would be critical for the comprehension of reversible passives because it provides the neural substrate implementing the working memory resources necessary to interpret complex linguistic structures. Re-analysis requires that information on the stimulus sentence be actively maintained while the outcomes of the first-pass sentence parsing are reviewed and/or revised. This account is clearly plausible from a cognitive point of view—noncanonical word order and passive voice complicate morphosyntactic structure, thereby increasing the need to maintain information in working memory for the time required by the re-analysis process. However, also this account is not fully supported by neuroanatomical data. Indeed, rTMS studies do suggest that inferior parietal regions provide the neural substrate of the storage component of working memory, but locate the critical areas anteriorly and laterally to the l-IPS (Romero et al. 2006; Papagno et al. 2007 and Romero Lauro et al. 2010). Furthermore, neuroimaging studies of the neural network involved in verbal working memory functions highlight the role of a fronto-parietal network that only partially includes the inferior l-IPS (Henson et al. 2000; Baldo and Dronkers 2006).

The present study narrows down the role of the l-IPS in sentence comprehension, by showing that this region plays a relevant role only when interpretation requires re-analysis, as in passive reversible sentences. However, results do not allow establishing if the l-IPS is critical because it is recruited when the phonological working memory load increases due to the co-occurrence of complex features such as passive diathesis and semantic reversibility, or when these features co-occur, irrespective of working memory demands. Both possibilities are only partially supported by extant data. In principle, the uncertainty could be solved by studies that successfully tease apart working memory and syntactic aspects of processing during the comprehension of passive reversible sentences.

## Conclusions

rTMS on the posterior l-IPS selectively affected RTs to passive reversible sentences, while leaving unaffected RTs to reversible active sentences and to irreversible sentences, both active and passive. This finding suggests that the co-occurence or the interaction of passive voice and semantic reversibility is critical for this effect, and confirms previous evidence supporting the role of the l-IPS in thematic role assignment. The specific contribution of this region to sentence interpretation remains elusive. The l-IPS could be involved in phonological working memory processes or it could be recruited by strictly linguistic phenomena. Distinguishing between the two alternatives is difficult, as sentence comprehension requires not only activating information stored in long-term memory (such as semantic and syntactic knowledge) but also keeping information on the to-be-processed stimulus active in working memory.
